# Circulating cytokine levels and antibody responses to human *Schistosoma haematobium*: IL-5 and IL-10 levels depend upon age and infection status

**DOI:** 10.1111/j.1365-3024.2010.01235.x

**Published:** 2010-11

**Authors:** T Milner, L Reilly, N Nausch, N Midzi, T Mduluza, R Maizels, F Mutapi

**Affiliations:** 1 Institute for Immunology & Infection Research, Centre for Infection Diseases, University of Edinburgh, Ashworth LaboratoriesEdinburgh, UK; 2 National Institutes for Health ResearchCauseway, Harare, Zimbabwe; 3 Department of Biochemistry, University of ZimbabweMount Pleasant, Harare, Zimbabwe

**Keywords:** antibody, Helminth, human, schistosomiasis, systemic cytokine

## Abstract

Experimental schistosome infections induce strong parasite-specific Th2 responses. This study aims to relate human systemic cytokine and antibody levels to schistosome infection levels and history. Levels of anti-*Schistosoma haematobium* antibodies (directed against crude cercariae, egg and adult worm antigens) and plasma cytokines (IFN-γ, IL-2, IL-4, IL-5, IL-10, IL-13, IL-17, IL-21, and IL-23) were measured by ELISA in 227 Zimbabweans (6–60 years old) in a schistosome-endemic area and related to age and infection status. Egg-positive people had significantly higher levels of specific antibodies, IL-2, IFN-γ and IL-23. In contrast, egg-negative individuals had significantly higher circulating IL-10, IL-4, IL-13 and IL-21 that were detected with high frequency in all participants. Subjects with detectable plasma IL-17 produced few or no eggs. When analyzed by age, IL-4 and IL-10 increased significantly, as did schistosome-specific antibodies. However, when age was combined with infection status, IL-5 declined over time in egg-positive people, while increased with age in the egg-negative group. Older, lifelong residents had significantly higher IL-4 and IL-5 levels than younger egg-negative people. Thus, a mixed Th1/Th2 systemic environment occurs in people with patent schistosome infection, while a stronger Th2-dominated suite of cytokines is evident in egg-negative individuals.

## Introduction

Schistosomes, commonly known as blood flukes are important trematode parasites of vertebrates and cause significant morbidity in humans. Of the three major human schistosome species, *Schistosoma mansoni* and *S. japonicum* adult parasites inhabit blood vessels of the intestine causing intestinal schistosomiasis, while *S. haematobium* adults are located in the bladder and pelvic plexuses causing urinary schistosomiasis. *S. haematobium* is the most prevalent species in sub-Saharan Africa, where it is responsible for a substantial amount of schistosome-associated pathology (1). Schistosome-specific acquired immunity capable of reducing levels of infection or re-infection develops slowly (2). The nature of these protective immune responses has been subject to intense analysis (3–6). Previous studies suggesting that anti-helminth immune responses fall into a Th1 (pro-inflammatory) and Th2 (anti-inflammatory) dichotomy with resistance to infection being associated with Th2 responses (7,8) failed to fully explain resistance/susceptibility to infection/re-infection in people resident in helminth endemic areas. For example, both Th1 and Th2 responsiveness appear compromised in schistosomiasis patients, and within the Th2 compartment, IL-5 responses are suppressed while IL-4 production is relatively intact (9). Further studies in Zimbabwe and Egypt showed no clear pattern between either Th1 or Th2 cytokine responses and infection intensity (10–12). More recently, studies characterising cellular responses have suggested the existence of a regulatory subset of T cells (Treg), which modulate the effects of Th1 and Th2 responses through the immunosuppressive cytokines interleukin-10 (IL-10) and transforming growth factor beta (TGF-β) (13) and have suggested that it is the balance between Th1, Th2 and Treg responses, which determines the outcome of helminth infections [reviewed in (14)].

The cytokine environment created during the development of helminth-specific immune responses is thought to have effects on unrelated antigens by promoting regulatory effector responses (15,16). Systemic cytokines are promiscuous rather than antigen specific in their effects (17). Thus, cytokines stimulated by helminth antigens can modify the environment in which responses to other pathogens occur as illustrated by the systemic effects of gut-restricted helminths (18,19). Currently, this paradigm is thought to explain the observed negative associations between helminth infections and atopic/autoimmune diseases (15,16,20,21).

Most previous cytokine studies in human schistosomiasis have focused either on parasite-specific recall responses or on a limited range of plasma cytokines (9,22). This study aims to investigate a comprehensive range of circulatory cytokine and antibodies and determine how these relate to the individual's current infection levels as well as to their history of exposure to schistosome infection. The cytokines measured were the pro-inflammatory cytokines IFN-γ, IL-17, IL-23 and the Th2-associated cytokines IL-4, IL-5, IL-10, IL-13 and IL-21 as well as IL-2.

## Materials and methods

### Study subjects

The study was conducted in two villages, Mutoko and Rusike in the Mashonaland East Province of Zimbabwe (31^°^30′E; 17^°^45′S), where *S. haematobium* is endemic. The participants have been involved in an ongoing study of the immunoepidemiology of human schistosomiasis (23,24). Ethical and institutional approval for the study was obtained from the Medical Research Council of Zimbabwe and the University of Zimbabwe, respectively. Permission to conduct the work in this province was obtained from the Provincial Medical Director. Prior to the study, informed consent was obtained from all participants or their guardians/parents in case of children. The villages were selected because the area has little or no other helminths and a low *S. mansoni* prevalence (<5%), and there had been no helminth control programmes in the area. Therefore, participants had not received any previous anti-helminthic treatment meaning that the natural immune responses could be studied in the absence of drug-altered schistosome responses (25). The main activity in these villages is subsistence farming; human water contact in rivers is frequent (at least 4 contacts/person/week) because of insufficient safe water sources and toilets.

### Parasitology and immunology samples

Stool and urine specimens were collected from each participant on 3 consecutive days and assayed for *S. haematobium, S. mansoni* and geo-helminths using standard procedures (26,27). *S. mansoni* and geo-helminth infections were detected from at least 4 Kato-Katz slides (maximum 6), 2 from each of the 2–3 stool samples collected and in addition, the formal ether concentration method was used as previously detailed (28,29) on 100 random samples to confirm the Kato-Katz results. Ten millilitres of venous blood was collected and processed as previously described (4) to obtain plasma for cytokine and antibody assays. Plasma was stored at −20°C and freighted frozen to Edinburgh where they were thawed for the first time for the assays described here. A thick smear slide was prepared upon blood collection for the microscopic detection of *Plasmodium* parasites. The inclusion criteria were that a participant had to (1) have provided at least two urine and two stool samples over 3 consecutive days, (2) be negative for intestinal helminths including *S. mansoni* (no one was excluded on this criteria as everyone was negative for these infections), (3) have given at least 10 mL of blood for serological assays and detection of *Plasmodium* parasites, (4) be lifelong residents of the area (assessed by questionnaire). Two hundred and twenty-seven people (112 men and 115 women) aged 6–60 met these criteria and formed our study population. All participants were offered anti-helminth treatment as a single treatment with praziquantel at the recommended dose of 40 mg/kg after surveying. Malaria cases were treated according to the treatment regime prescribed by the Ministry of Health in Zimbabwe.

### Antibody and cytokine assays

Circulating levels of IgA, IgE, IgG1, IgG2, IgG3, IgG4 and IgM directed against the three *S. haematobium* antigens cercariae (CAP), egg (SEA) and SWAP (adult worm) were detected by indirect enzyme-linked immunosorbent assays (ELISA) as previously described (30). Cytokine assays were conducted using capture ELISA with antibody sets and standards from BD Biosciences (IFN-γ, IL-2, IL-4, IL-5, IL-10, IL-13, IL-21, IL-23) and R and D Systems (IL-17) following previously published protocols (31) and manufacturer's guidelines. All assays were conducted in duplicate/sample with appropriate negative and positive controls for the antibody ELISA. People with cytokine levels above 0 ng/mL (after the subtraction of the blank control) were denoted positive for the cytokine and those with antibody levels above 3 standard deviations of the negative controls were denoted positive for the antibody.

### Statistical analyses

The number of people with detectable antibody/cytokine levels was compared between schistosome egg-negative and egg-positive people using a chi-squared test. Exploratory plots showed that the relationships between the different cytokines were nonlinear and directional (positive/negative), so correlation analyses were conducted using a one-tailed nonparametric Spearman correlation procedure (32).

The changes in cytokine levels with host age were analysed using a multivariate analysis with cytokine levels (square root transformed) as dependent variables and sex (categorical), village of residence (categorical) and host age (categorical) were the independent variables. Age was categorised into three groups; 5–10 years, 11–12 years and 13 years and above reflecting the epidemiological groupings (previously used for this population (4)) where schistosome infection levels are rising, peaking and declining, as well as to give comparable sample sizes for statistical analyses. The analysis used sequential sums of squares to allow for the effects of sex and village.

To determine the relationship between all the immunology measures and infection status, the 30 variables (9 cytokines + 21 antibody responses) were reduced into uncorrelated variables by a principal components analysis (PCA) procedure, a standard technique for reducing multivariate data to its main independent features by transforming several correlated variables into fewer uncorrelated variables called principal components (32). The components are extracted according to the amount of variation in the data they explain, so the first component explains the most variation, and each subsequent component is included if it explains a significant amount of variation present within the data (32). Principal components with eigenvalues greater than one were extracted by regression analysis, and an extracted component was considered reliable if it had two or more of the original variables with factor loadings ≥5 or ≤−5 and below. The relationship between infection status and the immunology variables (represented by the principal components) was tested using a generalised linear model procedure with the independent variables being sex (male, female), age group, residential areas (village 1 or 2) and principal components (continuous variables). Schistosome infection status denoted as negative for people with zero egg counts for all urine samples presented or positive for people with at least one egg detected in one of their urine samples was the dependent variable. All statistical analyses were performed using the SPSS software.

## Results

### Infection epidemiology

**Figure 1:**
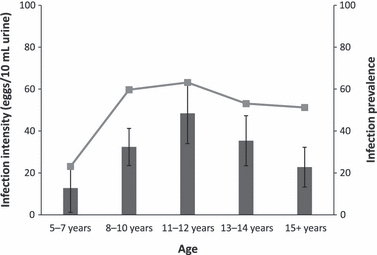
Age-associated *S. haematobium* infection intensity and prevalence. The intensity of infection was determined by arithmetic means of eggs/10 mL of urine in each of the 5 age groups. Bar graph represents infection intensity with standard error of the mean bars and line graph represents prevalence. Infection intensity followed a convex age-intensity pattern with participant age, while infection prevalence remained high after peaking in 11- to 12-year-old age group.

Overall, *S. haematobium* infection prevalence in the study population was 56% with a mean infection intensity of 34 eggs/10mL of urine (SEM = 6 eggs/10 mL urine, range 0–676 eggs/10 mL urine). Although these infection levels are moderate relative to reports from other areas in Zimbabwe (30), the World Health Organisation (WHO) denotes this prevalence level as high; infection intensity is also classified as high by the WHO definition of having more than 10% of the population with >50 eggs/10 mL of urine (45 of the 227 participants had >50 eggs per 10 mL urine) (33). Infection intensity rose to peak in childhood (11–12 years) followed by a sharp decline in infection intensity, while prevalence fell more gradually as shown in Figure 1. There is a low prevalence of nematode infections in rural Zimbabwe and consistent with a recent study (29), the prevalence of hookworm, *Ascaris lumbicoides* and *Trichuris trichiura* was zero in our study area. *Plasmodium falciparum* is the predominant species of malaria in Zimbabwe, and our study area is a mesoendemic area for malaria as detailed in (34) with a prevalence of 11% in our study population.

### Frequency of people with detectable antibody and plasma cytokine levels

All participants produced IgM antibodies directed against all the three schistosome life stages with the least prevalent antibody response being IgE (Table 1), indicating that they have all been exposed to schistosome antigens. When partitioned by infection status, a higher proportion of egg-positive people produced parasite-specific antibodies than egg-negative people (Table 1). This was significant for anti-cercariae IgE (*P* = 0·032, c^2^ = 4·233, df = 1) and IgG4 (*P* = 0·012, c^2^ = 6·1, df = 1), anti-worm IgG1 (*P* = 0·004, c^2^ = 8·12, df = 1) and IgG3 (*P* = 0·009, c^2^ = 6·44, df = 1) and anti-egg IgE (*P* < 0·001, c^2^ = 1 5·41, df = 1), IgG2 (*P* = 0·012, c^2^ = 6·52, df = 1) and IgG4 (*P* = 0·012, c^2^ = 6·52, df = 1). The presence of malaria parasites in the blood stream did not have a significant effect on the number of people with detectable plasma cytokine or antibody levels (analysis not shown).

**Table 1:** Number of people with detectable antibody levels directed against adult worm (anti-SWAP), cercariae (anti-CAP) and egg (anti-SEA)-soluble antigens partitioned by schistosome infection status

	S. *haematobium*			S. *haematobium*		
		
	Negative			Positive		
		
	Number with	Sample	% with	Number with	Sample	% with
						
	Detectable levels	Size	Detectable levels	Detectable levels	Size	Detectable levels
Anti-SWAP antibodies						
IgA	94	101	93	121	126	95
IgE	56	98	57	72	125	57
IgM	101	101	100	126	126	100
IgG1	89	101	88	123	126	93
IgG2	74	101	73	104	126	78
IgG3	77	101	76	112	126	83
IgG4	84	101	83	112	126	86
Anti-CAP antibodies						
IgA	97	101	96	123	126	98
IgE	84	101	83	116	126	92
IgM	101	101	100	126	126	100
IgG1	101	101	100	126	126	100
IgG2	100	101	99	124	126	98
IgG3	90	101	89	118	126	94
IgG4	83	101	82	117	126	93
Anti-SEA antibodies						
IgA	100	101	99	124	126	98
IgE	48	101	48	92	126	73
IgM	101	101	100	126	126	100
IgG1	99	101	98	126	126	100
IgG2	92	101	91	124	126	98
IgG3	97	101	96	124	126	98
IgG4	92	101	91	124	126	98

**Figure 2:**
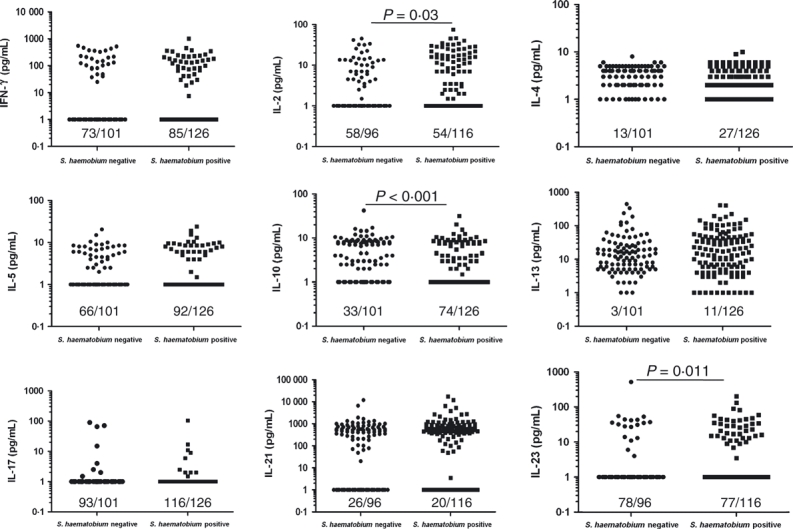
Systemic cytokine levels in the population partitioned by schistosome infection status. Bars represent the result of a chi-squared test comparing the number of people with detectable cytokine levels between schistosome egg-negative and egg-positive people. Figures represent the number of people in whom the cytokine was not detected out of the total number assayed who are either schistosome negative or positive.

Of the 227 participants, all but one person had at least one detectable cytokine. This individual was 11 years old and was negative for both schistosome eggs and malaria parasites. The least frequently detected cytokines were IL-17, IL-5 and IFN-γ, while the most frequently detected cytokines were IL-13, IL-4 and IL-21 (Table 2). Egg-positive people had more IL-2 and IL-23 producers compared to egg-negative people, although IL-23 positive people mostly carried light infections. More egg-negative people produced IL-10 compared to egg-positive people as shown in Figure 2.

**Table 2:** Number of people with detectable cytokine levels antigens divided by schistosome infection status. For each cytokine the number of people with detectable levels was compared between egg-negative and egg-positive people by a chi-squared (c^2^) test. Significant differences are denoted in bold

	*S. haematobium* negative			*S. haematobium* positive			Statistics
			
Cytokine	Number with detectable levels	Sample size	% with detectable cytokine levels	Number with detectable levels	Sample size	% with detectable cytokine levels	Egg+ve vs. egg−ve c^2^ value, *P*-value
IFN-gamma	28	101	28	41	126	33	0·62, *P* = 0·26
IL-2	38	96	40	62	116	53	**4·05, *P* = 0·03**
IL-4	88	101	87	99	126	79	2·88, *P* = 0·065
IL-5	35	101	35	34	126	27	1·56, *P* = 0·133
IL-10	68	101	67	52	126	41	**15·28, *P* < 0·001**
IL-13	98	101	97	115	126	91	3·21, *P* = 0·062
IL-17	8	101	8	10	126	8	0, *P* = 0·6
IL-21	70	96	73	96	116	83	3, *P* = 0·06
IL-23	18	96	19	39	116	34	**5·91, *P* = 0·011**

### Cytokine levels differ between hosts of different age

**Figure 3:**
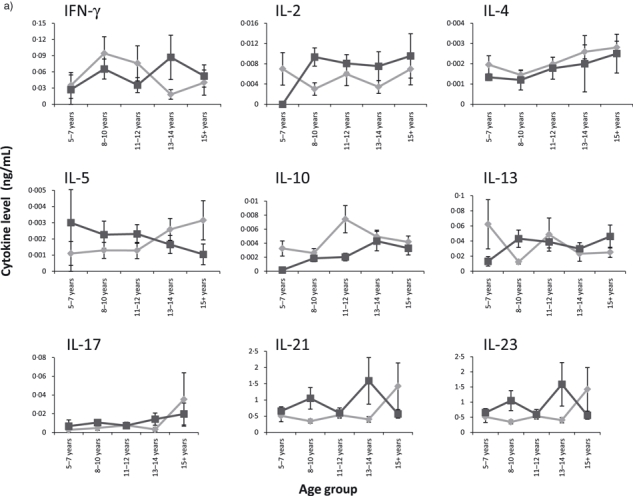

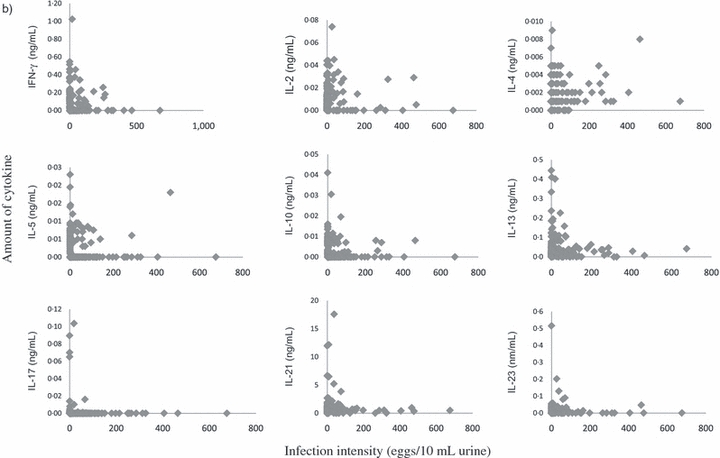
Age and infection intensity profiles of the cytokines. a) Relationship between cytokine levels and age-associated plasma cytokine levels partitioned by the participant's schistosome infection status (egg-positive vs. egg-negative) showing clear and distinct patterns for IL-4, IL-5 and IL-10. Squares = schistosome egg positive, diamonds = schistosome egg negative. b) Relationship between *S. haematobium* infection intensity for individual participants and their levels of plasma cytokines.

Levels of some cytokines varied with host age, the most pronounced changes occurring in IL-4 and IL-10 levels, which showed a significant overall increase with age (*F* = 21·9, df = 144·1, *P* < 0·0001 and *F* = 4·8, df = 144,1, *P* = 0·031, respectively). Further analyses of the cytokine age profiles partitioned by infection status indicated that there was large variation within cytokine levels between different age groups and consequently significant patterns could not be detected across the age range. However, Figure 3a shows that age-related changes in cytokine production partitioned by schistosome infection status were most pronounced for IL-4, IL-5, and IL-10. IL-5 levels declined with age in egg-positive people while IL-10 levels tended to increase. In egg-negative people IL-10 levels followed the age-infection profile while IL-5 levels lagged behind, only beginning to rise when infection levels peaked. IL-2, IL-13, IL-17, IL-21 and IL-23 showed little variation with host age in both egg-negative and egg-positive people. IFN-γ levels peaked early in egg-negative people but did not show a distinct peak in egg-positive people.

### Cytokine levels differ with schistosome infection intensity

Plots of infection intensity vs. cytokine levels indicated that most cytokines had a dichotomous relationship with infection intensity, so that high egg counts were associated with low cytokine levels. Notable exceptions were IL-4 and IL-2 as shown in Figure 3b, where there were some high cytokine levels associated with high egg counts. The 18 people with detectable IL-17 levels as well as the majority of IL-23 producers all carried little or no infection (none with more than 75 eggs/10 mL urine in the case of IL-17 producers). These relationships were not formally tested at this stage because of the dichotomous nature of the data but were included in the parametric tests determining the effect of systemic immune responses on schistosome infection intensity described later.

### Cytokine levels show correlations with each other

Levels of circulating cytokines were related to each other, with all the statistically significant associations being positive as shown in Table 3. IL-2 and IL-10 showed the most numerous significant associations with the other cytokines, while IL-21 showed the least association with any of the other cytokines measured (being related only to IL-2 and IL-23). Levels of IFN-γ (Th1 response marker), IL-4/IL-5 (Th2 response markers) and IL-17 (marker of Th17 responses) showed positive correlations with IL-10. IL-4 and IL-5 levels were significantly correlated despite the differences in the age profiles of their levels in egg-negative compared to egg-positive people described earlier. Similar to IL-23 levels, plasma levels of IL-4 and IL-5 were correlated to those of IL-17. The large number of significantly correlated cytokines measured in the study suggested potential underlying biological relationships. These relationships were captured in fewer variables (principal components), which were subsequently used to determine the effect of the potential underlying biological relationships on schistosome infection intensity.

**Table 3:** Cytokine correlations. Nonparametric Spearman's correlation between cytokines, giving the *r* coefficient and the *P*-value in brackets. Significant *P*-values are highlighted in bold while *P*-values still significant after Bonferoni correction (52) are indicated by shaded cells

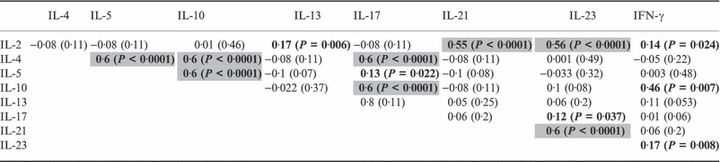

### Antibody and cytokine responses cluster into different immunological groups

The factor analysis reduced the 30 immunology variables into five main principal components (PCA) 1-5 described in Table 4. The PCA 1 is strongly influenced by the schistosome-specific antibody responses reflecting exposure and acquired immunity to the parasites. PCA 2 was made up of the cytokines IL-4 and IL-5 (Th2 cytokines) as well as some antibodies (anti-cercariae/IgM and IgG2 and anti-egg IgM previously associated with both exposure to and protection against infection) (35,36). PCA 3 was strongly influenced by levels of IL-10 and the antibodies anti-egg IgG1 and anti-adult worm IgE and IgA all of which have been associated with effector immune responses against the parasite (25,37–39). The two closely related cytokines IL-17 and IL-23 had the strongest loadings onto PCA 4, while IFN-γ and IL-2 had the strongest loading on PCA 5. Thus, the different principal components were taken to indicate different underlying biological process with PCA1 indicating exposure to the parasite and PCA 2-4 indicating different effector responses whose relationships to schistosome infection status were subsequently investigated.

**Table 4:** Principal components which are linear transformation of the large number of highly correlated antibody and cytokine variables into a smaller number of uncorrelated variables. The components (PCA1-5) are extracted according to the amount of variation in the data they explain so the first component explains the most variation and each subsequent component is included if it explains a significant amount of variation present with the data. The loading value of each individual cytokine or antibody indicates the correlation between the extracted component and the original variable so that variables with strong loadings (>5 or <−5) are indicated in bold

Immune variables measured	PCA1	PCA2	PCA3	PCA4	PCA5
% variation explained	28%	12%	10%	6%	**6%**
INF-y	0·11	−0·2	0·23	−0·08	**0·59**
IL-2	0·15	0·02	−0·05	0·1	**0·56**
IL-4	0·09	**0·69**	0·42	0·02	−0·01
IL-5	−0·01	**0·82**	0·04	0·08	−0·04
IL-10	0·01	0·38	**0·55**	−0·04	0·28
IL-13	−0·07	−0·01	−0·1	0	0·47
IL-17	0·08	0·17	0·21	**0·58**	0·08
IL-21	0·14	−0·16	0·1	0·41	0·33
IL-23	0·22	−0·09	0·24	**0·7**	0·27
CAP IgA	**0·7**	−0·1	0·37	−0·21	−0·12
CAP IgG1	**0·63**	0·35	−0·34	−0·01	0·19
CAP IgG2	0·4	−**0·5**	−0·22	0·27	−0·05
CAP IgG3	0·49	−0·42	−0·22	−0·23	0·25
CAP IgG4	**0·69**	−0·03	−0·23	0·32	−0·16
CAP IgE	**0·68**	−0·3	0·13	−0·24	−0·13
CAP IgM	**0·69**	0·53	0·02	−0·08	0·05
SEA IgA	**0·73**	−0·07	0·3	−0·17	−0·17
SEA IgG1	**0·64**	0·26	−**0·54**	−0·07	0·19
SEA IgG2	**0·59**	−0·47	−0·44	0·14	−0·1
SEA IgG3	0·45	−0·44	−0·21	−0·31	0·38
SEA IgG4	**0·68**	0·14	−0·4	0·09	−0·08
SEA IgE	**0·57**	0·13	0·05	−0·32	−0·17
SEA IgM	**0·65**	0·58	−0·14	−0·14	0·15
SWAP IgA	**0·54**	−0·23	**0·64**	0·08	−0·05
SWAP IgG1	**0·64**	0·33	−0·41	0·13	−0·04
SWAP IgG2	**0·57**	−0·21	0·27	0·4	−0·24
SWAP IgG3	**0·69**	−0·25	0·24	−0·1	0·02
SWAP IgG4	**0·62**	0·17	−0·21	0·3	−0·24
SWAP IgE	**0·55**	−0·25	**0·62**	−0·05	−0·04
SWAP IgM	**0·74**	0·21	0·34	−0·15	0·13

### Plasma cytokine and antibody responses vary with schistosome infection status

**Figure 4:**
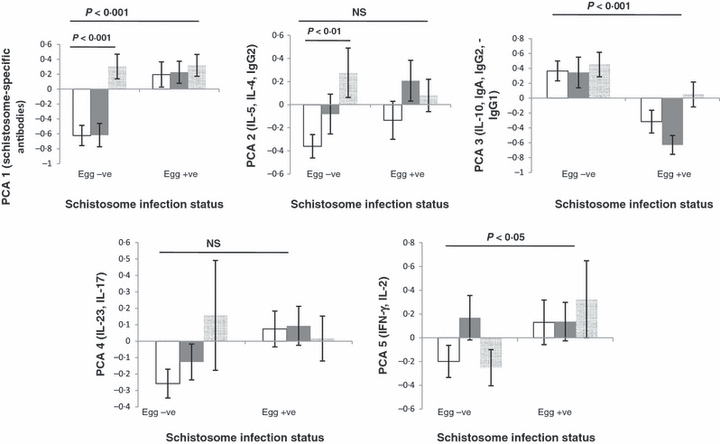
Relationship between systemic environment and schistosome infection status for the different participants divided by age group. Arbitrary values (*X*-axis) represent mean values of the principal components PCA1-5. Bars represent different age groups; open = 5–10 years old, solid = 11–12 years old and hashed 13+ years old. Statistical comparisons are made between egg-negative young people 5–10 years old (open bars) and old 13+ years old egg-negative people (hashed bars).

The effects of the principal components on schistosome infection status were determined after allowing for the effects of participant sex, residential village and age. This analysis showed that infection status was significantly associated with PCA1 (schistosome-specific antibody responses), PCA 3 (IL-10, anti-worm IgA, IgE and anti-egg IgG1) and PCA 5 (INF-γ and IL-2). In addition, the effects of PCA-5 on infection status were modified by PCA2 (IL-4, IL-5, CAP IgM and IgG2). PCA 4 (IL-17 and IL-23) did not have a significant effect on infection status shown in Table 5. As the study aimed to determine the effects of infection history on the systemic antibody and cytokines levels, PCA values in egg-negative people in the youngest age group (those with little or no experience of infection) were compared to those in the oldest age group (lifelong residence with little or no infection despite regular contact with infective water, putatively because of the development of acquired immunity). This analysis showed that levels of PCA 1 (antibodies) and PCA 2 (IL-4, IL-5, CAP IgM and IgG2) were significantly higher in older egg-negative people than in younger egg-negative people (Figure 4). There were no significant differences amongst the other PCAs. The analysis also showed some interesting though not significant differences between the youngest and oldest egg-positive people where levels of both PCA 2 and PCA 3 were higher in the oldest compared to the youngest age group (Figure 4).

**Table 5:** Relationship between systemic immune responses (antibody and cytokine) and *S. haematobium* infection status. The test statistics were obtained using sequential sums of squares which tested for each variable after allowing for all the variables preceding it in the model. The variables were tested in the order in which they are presented in the table, after allowing for participant sex, village of residency and age group. Schistosome infection status was denoted as negative for people with zero egg counts for all urine samples presented or positive for people with at least one egg detected in one of their urine samples was the dependent variable. Significant variables are shown in bold.

Source of variation	df	Mean square	*F*	*P*-value
PCA1(antibody responses)	**1**	**3·64**	**19·29**	**0·00**
PCA2(IL-4, IL_5, CAP IgM, IgG2)	1	0·33	1·73	0·19
PCA3(IL-10, -SEA IgG1, SWAP IgA, SWAP IgE)	**1**	**4·50**	**23·85**	**0·00**
PCA4(IL-17, IL-23)	1	0·12	0·61	0·43
PCA5(IFN-y, IL-2)	**1**	**1·09**	**5·79**	**0·02**
PCA1*PCA2	1	0·69	3·64	0·06
PCA1*PCA3	1	0·06	0·33	0·57
PCA1*PCA4	1	0·47	2·47	0·12
PCA1*PCA5	1	0·06	0·30	0·58
PCA2*PCA3	1	0·10	0·53	0·47
PCA2*PCA4	1	0·00	0·00	0·95
PCA2*PCA5	**1**	**0·89**	**4·72**	**0·03**
PCA3*PCA4	1	0·22	1·15	0·28
PCA3*PCA5	1	0·15	0·77	0·38
PCA4*PCA5	1	0·00	0·00	0·95
Total	191			

## Discussion

This study focused on systemic cytokine and antibody responses in Zimbabweans exposed to *S. haematobium* infection to determine the relationship between host immune responses and schistosome infection status. Several experimental and human studies investigating schistosome-specific responses have described the presence of schistosome-specific Th2 responses (40).This study investigated whether these parasite-specific responses were reflected in the systemic environment, representing both broader immune activation to pathogen and environmental challenges, and immunological cross-talk which may down-regulate particular components.

Not surprisingly, all participants had detectable levels of parasite-specific antibodies particularly IgM reflecting that they had been exposed to schistosome infection. Similarly, all participants except one egg-negative child had detectable levels of at least one cytokine. The systemic environment in schistosome egg-positive participants differed from that in egg-negative participants, and differed between young and old egg-negative people (i.e. those with a short vs. long history of exposure to schistosome infections).

Systemic cytokines are produced in response to different antigens arising from various exogenous sources but despite the potentially large number of cytokine-inducing agents, there was a significant relationship between circulating cytokines and schistosome infection status. Significantly more schistosome egg-negative individuals had detectable levels of IL-10 than egg-positive people, whereas a significantly larger number of egg-positive people had detectable IL-2 and IL-23 levels. While the role of IL-23 in human schistosomiasis remains to be established, its more frequent occurrence in egg-positive participants is consistent with its postulated role in immunopathology suggested by murine studies (41). In murine schistosomiasis (41), IL-23 and IL-17 are associated with the development of pathology. In this study, the two cytokines were positively correlated, but the 18 people with detectable IL-17 were mostly egg-negative or people excreting low levels of schistosome eggs (people who are less likely to have egg-induced immunopathology), the most heavily infected people (people who would be expected to have egg-induced immunopathology) did not have detectable levels of IL-17. The two cytokines IL-13 and IL-4 were produced by a large number of the study participants, but they showed no relationship to schistosome infection status either alone or in the PCA analysis. They may have been stimulated in response to other exogenous agents such as other pathogens (e.g. HIV (42) which is prevalent in Zimbabwe) and allergens (43) (e.g. house dust mites) not investigated in this study.

Interestingly, despite the overall significant positive correlation between IL-4 and IL-5 levels, the age profiles of the two cytokines differed. IL-4 levels increased with age across the population but, in contrast, IL-5 levels increased with age only in egg-negative people but declined with age in egg-positive people. This suggests that there may be temporal differences in the production of the two cytokines with respect to antigen exposure. Field studies in schistosomiasis (9,12) and filariasis (44) as well as mechanistic experimental studies (45) have reported dissociation in IL-4 and IL-5 production and effector functions. Overall, the systemic levels of IL-4, IL-5, IL-10 and IFN-γ showed similar patterns with age to those previously reported for the parasite-specific cytokines in this population (46), indicating that schistosome-specific cytokine responses are associated with systemic cytokine levels.

The study clearly demonstrated that the systemic antibody and cytokine levels clustered into different groups, reflecting exposure to parasite antigens as well as different effector-related responses. Parasite-specific antibody responses mostly clustered together and were higher in egg-negative people compared with egg-positive people. These antibody responses would represent both responses indicating exposure to infection as well as responses associated with protection to infection/re-infection. The Th2-like cytokines together with anti-cercariae IgM and IgG2 were also higher in egg-negative people, but their association with infection status was modified by the levels of IFN-γ and IL-2 consistent with Th1/Th2 cross-regulation of immune responses (47). IL-10 and the antibodies IgG1, IgA and IgG4 were significantly higher in egg-negative than egg-positive people. Comparison of systemic levels of antibodies and cytokines in the youngest and oldest egg-negative participants allowed the effect of differences in history of exposure to parasites antigens to be determined. The use of age as a proxy for exposure history is possible in this population because all participants were lifelong residents of the area with frequent contact with infective water and had never received anti-helminthic treatment. This analysis showed a clear difference in levels of parasite-specific antibodies and levels of the Th2 cytokines (IL-4 and IL-5), both of which were significantly higher in older egg-negative people suggesting that these were associated with resistance to infection/re-infection.

This study indicates that *S. haematobium* infection is associated with a Th2-systemic response, but that this response differs between people carrying a patent infection (positive egg counts) and putatively resistant individuals (egg-negative lifelong residents with continued exposure to infective water) who have experienced previous infection (as evidenced by the presence of schistosome-specific IgA, IgG and IgE antibodies). Egg-positive people have a modified Th2 systemic environment composed of parasite-specific antibodies and IL-4 which is modulated by IFN-γ and IL-2, and levels of the potentially protective IL-5 decline over time. They also have a more prevalent IL-21 and IL-23 response than egg-negative people, suggesting that either or both Th1 or Th17 cells are counteracting the development of a strong Th2 response. In addition, IL-2 is a key cytokine for the maintenance of regulatory T cells (48), which may show heightened activity in patent infection (49). Older egg-negative people have a fully Th2-dominated cytokine environment comprising of IL-4, IL-5, IL-10 and parasite-specific antibodies. It is particularly interesting that IL-10 is indicated to be primarily a pro-Th2 factor, rather than playing an immunosuppressive role. Analysis of the peripheral blood mononuclear cells from the population will allow us to determine whether in human schistosomiasis, as recently reported in filariasis, the majority of IL-10 production derives from CD25-negative Th2-like cells, rather than the regulatory T cell subset (50).

In conclusion, the systemic cytokine environment in schistosome exposed/infected people does reflect the infection status of the host, and varies significantly with age i.e. history of exposure to infection. Furthermore, a mixed Th1/Th2 systemic environment occurs in people with patent schistosome infection, while a stronger Th2-dominated suite of cytokines is evident in egg-negative individuals. Future studies integrating our understanding of both total and antigen-specific cytokine production will help to further identify the key components of anti-parasite immunity and to develop possible mechanistic explanations for the helminth-associated immunomodulation of responses to third party antigens (51).
